# Letter to the editor: Migrant tuberculosis in low-incidence Japan; introduction of pre-entry screening

**DOI:** 10.2807/1560-7917.ES.2025.30.25.2500404

**Published:** 2025-06-26

**Authors:** Mugen Ujiie, Akihiro Ohkado, Tomohiko Ukai, Norio Ohmagari, Seiya Kato

**Affiliations:** 1 Disease Control and Prevention Center, Japan Institute of Health Security, Tokyo, Japan; 2 The Research Institute of Tuberculosis, Tokyo, Japan; 3 Japan Institute for Health Security, Tokyo, Japan

**Keywords:** Tuberculosis, Migration, Pre-entry screening, Low-incidence country, Japan

**To the editor**: We read with great interest the recent study by Domaszewska et al. on tuberculosis (TB) rates among migrants in low-incidence European countries [1]. Their study highlights the considerable contribution of foreign-born populations to the domestic TB incidence and the importance of targeted screening and preventive measures. Here, we present an update on the TB epidemiology in Japan and recent policy developments that address these issues.

In 2021, Japan’s national TB notification rate dropped to 9.2 per 100,000 (11,519 cases) [2], passing below the threshold defining a low-incidence country (< 10 per 100,000) [3]. This achievement was accompanied by a clear epidemiological shift, with foreign-born individuals accounting for an increasing share of TB notifications. During that year, with over 95% of notified cases having their country of birth recorded (i.e. 96.6%; 11,122/11,519), the proportion of foreign-born cases among total TB notifications was 11.4% (1,313/11,519); in the 15–24-year age group (n = 538), it reached 68.4% (n = 368) [2]. Furthermore, in 2023, 1,619 (16%) of the 10,096 new TB cases — birthplace known for 9,825 (97.3 %) — were foreign-born [4], and 87.8% (534/608) of newly notified TB cases in the 15–24-years-old age group were foreign-born. This reflected profound changes in TB epidemiology, with the increase in foreign-born cases contributing to the stagnation of the downward trend of TB annual incidence in Japan and raising major public health concerns [5].

In response, Japan introduced a pre-entry TB screening programme (JPETS) for long-term visa applicants from the top six countries, from which the highest numbers of TB cases have been detected in Japan. The official operations were launched on 24 March 2025 [6]. The first rollout covers Nepal, the Philippines, and Viet Nam and will expand to China, Indonesia, and Myanmar/Burma in due time. These six countries account for 83.5% of foreign-born TB cases (1,352 of 1,619) in Japan in 2023 [4]. Notably, just over half of foreign-born TB cases are diagnosed within the first 2 years after arrival in Japan, supporting the value of early detection [4]. The JPETS initiative aims to identify and treat active TB in prospective entrants before they travel to Japan, thereby protecting both migrants and the host community.

Japan’s adoption of pre-entry TB screening aligns with practices in some other countries with a low incidence. Australia, Canada, New Zealand, the United Kingdom, and the United States mandate pre-immigration TB testing for entrants from high-incidence countries/areas [7]. By joining these countries to implement screening abroad, Japan has strengthened its domestic TB control through a preventive border strategy. Pre-entry screening is expected to reduce the importation of active TB and support the progress towards elimination [7]. However, as previous analyses have emphasised, this initiative also requires robust health-information systems, coordination among agencies, and ongoing monitoring and evaluation [8]. Importantly establishing a pre-entry screening programme is always the most difficult step and should not be underestimated [8].

Screening alone is insufficient for controlling TB among migrants in the long term. It must be combined with post-arrival surveillance, timely access to care, and interventions for latent TB infection (LTBI) to maximise its effectiveness [8]. Japan’s experience as a new low-incidence country underscores the need to continually adapt TB control strategies to address its evolving epidemiology. As highlighted in the European context, addressing TB in migrant populations is crucial to further reduce the impact of disease in low-incidence settings [1]. Ongoing surveillance, international collaboration, and investment in health-information systems are all essential for evaluating and refining these measures [8]. We believe that Japan’s evolving TB strategy will be of broad interest and will reaffirm the importance of targeted screening in the effort towards TB elimination. 
